# Supplementary material for: Exosome-mediated apoptosis pathway during WSSV infection in crustacean mud crab
Source: PLoS Pathog. 2020 May 20;16(5):e1008366. doi: 10.1371/journal.ppat.1008366 (PMC7266354; doi:10.1371/journal.ppat.1008366)
Supplement: Attachment — Submitted filename: Response to the decision letter.doc [file ppat.1008366.s001.doc]

**Response to the decision letter**

Editors

PLOS Pathogens

Date: Apr 28, 2020

Dear Editors,

We are pleased to know that our manuscript entitled “Exosome-mediated apoptosis pathway during WSSV infection in crustacean mud crab” has the opportunity to be resubmitted. We would like to take this opportunity to appreciate your time and to thank the anonymous reviewers for their valuable suggestions and comments. The manuscript has been carefully revised and greatly improved for additional related experiments, data analysis and language writing, accordingly. All changes are highlighted with red color in the revised manuscript. The point-to-point answers to the comments of the reviewers are as follows.

**For Reviewer 1:**

**Q1:** The manuscript contains some ill sentences and grammar errors. Addressing these concerns would help them to improve their manuscript. For example, the Latin name spelling should be *Scylla paramamosain*, not *Scally paramamosain*.

**Answer:** As suggested, two native English-speakers have already edited the entire manuscript. We believe it has significantly improved for the language issues raised by the reviewers.

**Revisions in the revised manuscript:** The entire manuscript.

**Q2:** In references section, article titles should only be capitalized on the first word, not the first letter of each word.

**Answer:** As suggested, the references section is modified in the revised manuscript.

**Revisions in the revised manuscript:** The references section.

**Q3:** The protocols of exosomes isolation should be described in detail. At present, there is no mature instruction for the exosomes isolation in crustaceans.

**Answer:** As suggested, the protocols for exosomes isolation in the Materials and Methods section are modified and described more detailed in the revised manuscript.

**Revisions in the revised manuscript:** Lines 415-424.

**Q4:** The authors found that two exosomal miRNAs (miR-137 and miR-7847) were both involved in antiviral immune regulation in mud crab. It is necessary to explore whether there exist primary or secondary relationships during the regulatory process.

**Answer:** We want to thank the reviewer for the excellent suggestion. To solve this problem, mud crabs were co-injected with either exosome-PBS, exosome-PBS and AMO-miR-137, exosome-PBS and AMO-miR-7847 and WSSV. The results showed that both miR-137 and miR-7847 silencing could suppress virus replication separately. Thus, combining with the data shown in Fig. 3G, we speculated that there existed no primary or secondary relationships during the regulatory process, both miR-137 and miR-7847 could function separately.

**Revisions in the revised manuscript:** Fig. 3H; Lines 191-197; Lines 760-763.

**Q5:** The authors only detect the interactions between miRNAs and AIF, while the relationship between exosomes and AIF is not studied. Thus, the effect of exosomes challenge on AIF expression should also be investigated.

**Answer:** As suggested, the isolated exosomes from mud crab with different treatments (including PBS and WSSV) were injected into mud crab, and then the expression level of AIF was detected via western blot, the results showed that the expression of AIF was significantly increased in the exosome-WSSV treated mud crabs compared with the control.

**Revisions in the revised manuscript:** Fig. 5E; Lines 245-246; Lines 791-794.

**Q6:** It is found from the data that AIF could interact with HSP70, while HSP70 could interact with Bax. Thus, whether AIF could directly interact with Bax should be clarified in detail accordingly.

**Answer:** As suggested, to confirm whether AIF could bind to Bax directly, the interactions between AIF and Bax was investigated, the cell lysates were subjected to Co-IP assay with anti-AIF IgG and anti-Bax IgG, the results showed that AIF could not IP with Bax, while Bax also could not IP with AIF, thus, we speculated that HSP70 formed complex with AIF and Bax separately.

**Revisions in the revised manuscript:** Fig. 7G; Lines 276-280; Lines 821-824.

**For Reviewer 2:**

**Q1:** It is not clear what is the ratio of EVs to Cells, i.e., Is it 1:1010, 1:1000, 1:10000 to score for an effect?

**Answer:** Sorry for make the confusion in our previous manuscript. In this study, the effects of exosomes are measured *in vivo* (mud crab), since the number of cells in mud crab is difficult to estimate. Therefore, in order to provide a reference standard, we attempt to evaluate the ratio of exosomes to the bodyweight of mud crab. The ratio is 2×10^7^ vesicles/50g mud crab, the relevant information is added in the revised manuscript.

**Revisions in the revised manuscript:** Lines 428-430.

**Q2:** Data in Figure 2D requires at least multiple WBs to validate the results with confidence.

**Answer:** As suggested, the experiment in Figure 2D was re-conducted, we used two proapoptotic proteins (BAX and p53) and two pro-survival proteins (Bcl-2 and BAG1) to validate the results.

**Revisions in the revised manuscript:** Fig. 2D; Lines 146-148.

**Q3:** For mir137 to work in cells, they require high levels of miRNA machinery components. What are the levels of Ago2, Dicer, TRBP, *etc*. in the recipient cells?

**Answer:** It is a good suggestion. To address this issue, the isolated exosomes from mud crabs with different treatments (including PBS and WSSV) were injected into mud crabs, then the expression levels of the indicated genes (Ago2, Dicer and TRBP) were detected via qPCR. However, the results showed that Ago2, Dicer and TRBP were kept unchanged in the exosome-PBS and exosome-WSSV treated mud crabs. Maybe the reason is that the miRNA machinery components work for all the miRNAs in mud crab, but not some of the specific miRNAs (References 1-2). While through miRNA microarray, we found that miRNA abundance in exosome-PBS and exosome-WSSV treatments did not significantly changed (some miRNAs were upregulated, some miRNAs were downregulated, some miRNAs kept unchanged). Thus, this might lead Ago2, Dicer and TRBP to be kept unchanged in the exosome-PBS recipient cells and exosome-WSSV recipient cells (**Fig.1**).

The primers used and qPCR data were list below:

Ago2-F: 5’-GCGGGTGACAACAAGAAACC-3’,

Ago2-R: 5’-CGTGCCCTGAATACCTTGGT-3’,

Dicer-F: 5’-CGAGAGTTGCCCACAGACTT-3’,

Dicer-R: 5’-GACAGAAAGGGCCTGACACA-3’,

TRBP-F: 5’-CGGCAGCTATTGAGGGCATA-3’,

TRBP-R: 5’-GCCAGTTTCTTGCTCTTGCC-3’.

Fig.1 Ago2, Dicer and TRBP were unchanged in exosome-PBS and exosome-WSSV recipient cells

**Q4:** What percent of HSP70 binds to AIF vs Bax?

**Answer:** As suggested, to measure the percent of HSP70 binds to AIF vs Bax, we performed Co-IP assay with anti-AIF IgG and anti-Bax IgG, then the IP products were subjected to western blot analysis and Gray-scale measurement via Image J software. The results showed that about 18.55% HSP70 was bound to AIF, while about 81.45% HSP70 was bound to Bax.

**Revisions in the revised manuscript:** Fig. 7H; Lines 280-284; Lines 824-827.

I think that the revision would meet the reviewers’ comments and suggestions and our manuscript now is suitable for resubmission. In the case of any questions, please feel free to contact me.

References

1.Tang G. siRNA and miRNA: an insight into RISCs[J]. Trends in Biochemical Sciences, 2005, 30(2):106-114.

2.Stroynowska-Czerwinska A, Fiszer A, Krzyzosiak WJ. The panorama of miRNA-mediated mechanisms in mammalian cells[J]. Cellular & Molecular Life Sciences, 2014, 71:2253–2270.

Thank you very much!

Yours sincerely,

Shengkang Li

College of Sciences, Shantou University

China
